# The Contribution of Copy Number Variants and Single Nucleotide Polymorphisms to the Additive Genetic Variance of Carcass Traits in Cattle

**DOI:** 10.3389/fgene.2021.761503

**Published:** 2021-11-02

**Authors:** Pierce Rafter, Isobel Claire Gormley, Andrew C. Parnell, Saeid Naderi, Donagh P. Berry

**Affiliations:** ^1^ Animal & Grassland Research and Innovation Centre, Fermoy, Ireland; ^2^ School of Mathematics and Statistics, University College Dublin, Dublin, Ireland; ^3^ Hamilton Institute, Maynooth University, Maynooth, Ireland; ^4^ Irish Cattle Breeding Federation, Bandon, Ireland

**Keywords:** CNV, Charolais, Holstein-Friesian, Limousin, SNP, LASSO

## Abstract

The relative contributions of both copy number variants (CNVs) and single nucleotide polymorphisms (SNPs) to the additive genetic variance of carcass traits in cattle is not well understood. A detailed understanding of the relative importance of CNVs in cattle may have implications for study design of both genomic predictions and genome-wide association studies. The first objective of the present study was to quantify the relative contributions of CNV data and SNP genotype data to the additive genetic variance of carcass weight, fat, and conformation for 945 Charolais, 923 Holstein-Friesian, and 974 Limousin sires. The second objective was to jointly consider SNP and CNV data in a least absolute selection and shrinkage operator (LASSO) regression model to identify genomic regions associated with carcass weight, fat, and conformation within each of the three breeds separately. A genomic relationship matrix (GRM) based on just CNV data did not capture any variance in the three carcass traits when jointly evaluated with a SNP-derived GRM. In the LASSO regression analysis, a total of 987 SNPs and 18 CNVs were associated with at least one of the three carcass traits in at least one of the three breeds. The quantitative trait loci (QTLs) corresponding to the associated SNPs and CNVs overlapped with several candidate genes including previously reported candidate genes such as *MSTN and RSAD2,* and several potential novel candidate genes such as *ACTN2* and *THOC1*. The results of the LASSO regression analysis demonstrated that CNVs can be used to detect associations with carcass traits which were not detected using the set of SNPs available in the present study. Therefore, the CNVs and SNPs available in the present study were not redundant forms of genomic data.

## Introduction

Carcass value is an important component of both beef-on-beef (Connolly et al., 2016) and beef-on dairy (Berry et al., 2019) breeding objectives. The use of genomic data in estimating breeding values for cattle has the potential to further increase the efficiency of beef breeding strategies by shortening the generation interval thereby accelerating genetic gain (Meuwissen et al., 2001). Genomic predictions and genome-wide association studies in cattle tend to use single nucleotide polymorphism (SNP) data, and increasingly (imputed) whole genome sequence, as the genomic features (Gutierrez-Reinoso et al., 2021). While multiple genome-wide association studies of carcass traits in cattle have successfully identified genomic regions associated with carcass metrics using SNP genotype data (de Oliveira Silva et al., 2017; Hay and Roberts, 2018; Purfield et al., 2019), SNP genotypes are not the only type of genomic data that can be derived from SNP arrays. Copy number variants (CNVs) are a type of genetic variant formed by deletion or duplication of genomic DNA (Feuk et al., 2006); typically CNVs are defined to have a minimum genomic length of between 50 bp (Mills et al., 2011) and 1 kb (Feuk et al., 2006). Duplications or deletions below the minimum genomic length threshold of a CNV are usually referred to as insertion or deletions (InDels) (Werdyani et al., 2017). Several CNV association analyses in cattle have identified CNVs associated with important traits, including meat quality traits (da Silva et al., 2016), carcass merit (Zhou et al., 2016), and milk yield (Xu et al., 2014; Prinsen et al., 2017). While these CNV association analyses have identified genomic regions associated with important traits in cattle using only CNV data, it is not always clear whether those same genomic regions could have been detected using another type of genetic marker such as SNPs. A fundamental principle of genome-wide association studies is that linkage disequilibrium exists between the genetic markers and the causal mutation(s) affecting the trait of interest (Balding et al., 2006). Adding CNV information to existing SNP genotype data in genomic evaluations or genome-wide association analyses may not contribute any new information if the CNVs are in strong linkage disequilibrium with genotyped SNPs. Previous studies estimate that approximately 75% of deletion CNVs are in linkage disequilibrium with flanking SNP genotypes (Xu et al., 2014; Hay et al., 2018). However, imputation analyses demonstrated that CNVs cannot be consistently imputed with high reliability from flanking SNP haplotypes (Handsaker et al., 2015; Rafter et al., 2020); this implies that CNVs are frequently not in linkage disequilibrium with flanking SNP haplotypes. Given that there is some degree of incomplete linkage between CNVs and SNP genotypes, it could mean that genomic analyses that only use SNP genotypes as genetic markers may not detect associations where the causal variant is a CNV. Hay et al. (2018) carried out the first genomic predictions of carcass traits in cattle using both SNP and CNV data and found that, for some traits, exploiting CNV information increased the accuracy of the genomic predictions. While this study by Hay et al. (2018) did not directly calculate the additive genetic variance accounted for by CNVs, it suggests that CNVs do account for some of the additive genetic variance of carcass traits which cannot be accounted for by SNP data.

The objective of the present study was to quantify the relative contribution of CNV data and SNP genotype data to the additive genetic variance of carcass traits in cattle. A secondary objective was to jointly evaluate SNP genotypes and called CNVs in a least absolute selection and shrinkage operator (LASSO) regression model to identify genomic regions associated with carcass traits in cattle.

## Materials and Methods

### Genotype Data

All animals in the dataset were genotyped on the Illumina BovineHD (Illumina Inc. San Diego, CA) SNP genotype array (777,962 SNPs). The chromosome and positions of the SNPs were taken from the UMD3.1. (Zimin et al., 2009) assembly of the cattle genome. Animals which had less than 95% of their SNPs called were discarded from the dataset. Similarly, SNPs with a call-rate less than 95% were discarded, as were SNPs on the X and Y chromosomes, or SNPs without a recorded chromosome or position. A further 1,611 SNPs that deviated from expected Mendelian inheritance patterns in more than 2% of parent-progeny pairs (Purfield et al., 2015) were also discarded from the final dataset; these SNPs were detected using the 1,477 parent-progeny pairs in the dataset. After genotype edits, the dataset consisted of 1,324 Holstein-Friesians, 981 Charolais, and 1,129 Limousins, each with 712,555 SNPs.

### Phenotype Data

Estimated breeding values (EBVs) for carcass weight, carcass fat, and carcass conformation were obtained for each genotyped animal in the dataset from the January 2019 national genetic evaluation of the Irish Cattle Breeding Federation (ICBF) database (Bandon, Co. Cork). Each of the animals in the present study were sires. The EBVs of these sires were estimated from their descendants; the EBVs of the sires were the equivalent to >150,000 effective phenotypic records. Carcass weight is the weight of the animal, in kg, after organs, visceral fat, limbs, and head have been removed (Englishby et al., 2016). Carcass fat and carcass conformation scores were obtained from video imaging analysis; these scores are based on the 15-point EU beef classification system (Pabiou et al., 2011). The EBVs of each of the three carcass traits were deregressed using Mix99 software (Stranden and Lidauer, 1999) using the Secant method (Stranden and Mantysaari, 2010). The effective record contribution of each animal in the population was calculated using the method described by Harris and Johnson (1998). Animals with an effective record contribution of <1 were excluded from the final dataset. The number of animals available in each breed for each of the three carcass traits after edits is in Table 1.

**TABLE 1:** The number of animals and copy number variants (CNVs) available for each breed and trait along with the number of SNPs and CNVs associated with each of the three carcass traits within breed, and the percentage of the genetic variance accounted for by the associated CNVs identified by the LASSO regression analysis for each breed and trait.

Breed	Trait	Sample size	Number of CNVs	Associated SNPs	Associated CNVs	Variance explained by CNVs (%)
Charolais	Weight	945	3,954	116	2	1.236
Charolais	Fat	945	3,954	254	9	1.142
Charolais	Conformation	945	3,954	218	3	0.101
Holstein-Friesian	Weight	892	13,899	0	0	0.000
Holstein-Friesian	Fat	923	13,953	22	0	0.000
Holstein-Friesian	Conformation	915	13,969	49	2	0.645
Limousin	Weight	974	2,805	11	0	0.000
Limousin	Fat	973	2,804	254	1	0.091
Limousin	Conformation	974	2,805	63	1	0.261

### Copy Number Variant Detection

Copy number variants were called from the edited high-density SNP array (712,555 autosomal SNPs) data using PennCNV (Wang et al., 2007) and QuantiSNP (Colella et al., 2007). PennCNV and QuantiSNP were both used separately to call CNVs from each animal in the population; CNVs called by either PennCNV or QuantiSNP which contained at least 3 SNPs were retained in the dataset. Diskin et al. (2008) reported that the guanine-cytosine (GC) content of DNA biases CNV detection from SNP array data. An adjustment for GC bias in CNV detection was applied by both QuantiSNP and PennCNV, the GC content of the genome was calculated using the UMD3.1 assembly of the cattle genome, compiled as of June 2014. A CNV was considered to have been called by both PennCNV and QuantiSNP when the endpoints of the CNV called by one of the algorithms was within 1 SNP of the endpoints called by the other algorithm. A single SNP difference in endpoint identification between PennCNV and QuantiSNP was allowed for because if there is a difference between the true endpoint of a CNV and the called endpoint, the called endpoint is typically only a single SNP from the true endpoint of the CNV (Colella et al., 2007; Dellinger et al., 2010). Copy number variants called by either PennCNV or QuantiSNP were included in the dataset; CNVs called by both algorithms from the same animal were not double counted. Finally, CNVs had to be present in at least 3 animals within breed to be included in the final dataset. The final number of CNVs available for each breed and trait after edits is in Table 1.

### Genomic Relationship Matrix

Genomic relationship matrices (GRMs) were calculated separately for the CNV data and the SNP genotype data within each of the three breeds using method 1 described in VanRaden (2008):

$$\mathrm{GRM}=\frac{\left(M-P\right){\left(\mathrm{M-P}\right)}^{T}}{2\sum p_{i}\left(1-p_{i}\right)}$$

where **M** is the matrix of genetic marker genotypes recoded as -1, 0, 1 representing the homozygote, heterozygote, and the other homozygote, respectively, **P** is a matrix of allele frequencies for each genetic marker in the dataset, i^th^ column vector of **P** is given by 2( $p_{i}-0.5)$ where $p_{i}$ is the frequency of the second allele for the *i*th genetic marker. Prior to the calculation of the SNP-derived GRMs, any missing genotypes were imputed within breed using the imputation software FImpute (Sargolzaei et al., 2014).

For each breed, the CNV-derived GRM was calculated twice using two separate procedures. In the first procedure, double-deletions and double-duplications were recoded as -1, single-deletions and single-duplications were recoded as 0, and the normal state (i.e. the absence of a CNV) was recoded as 1. In the case of mixed-CNVs (CNVs which are present as both deletions and duplications in the population), in order to avoid treating deletions and duplications as identical variants, each mixed-CNV was treated as two distinct loci, one to represent the deletions and the other to represent the duplications. For the deletion locus, duplications were recoded as normal state (i.e. no CNV); similarly for the duplication locus, any deletions were recoded as normal state. In the second procedure to calculate the CNV-derived GRM, mixed-CNVs were not treated as two distinct loci but instead all deletions were recoded as -1, the normal state was recoded as 0, and all duplications were recoding as 1.

### Population Structure

A GRM of the entire population (i.e. the combined Charolais, Holstein-Friesian, and Limousin populations) was constructed using the imputed SNP genotype data (712,555 SNPs) for all available animals. Principal components analysis of this GRM was used to determine population substructure within the entire population of available animals (Patterson et al., 2006; Kelleher et al., 2017). Separately, principal components analysis was also carried out individually on the SNP-derived GRMs for the Charolais, Holstein-Friesian, and Limousin populations.

### Variance Components Analysis

To calculate the relative contribution of CNV data and SNP genotype data to variance in the deregressed EBVs for each animal in the population, the variance explained by a SNP-derived GRM and the variance explained by a CNV-derived GRM were jointly considered in a linear mixed model. The fitted model was:

dEBV=μ+Za+Wb+e

where **dEBV** was a vector of deregressed EBVs of each animal in the population. The intercept of the model was denoted by **μ** and was treated as a fixed effect**,** the vector **a** was a random effect with distribution N(0, $G_{SNP}\sigma_{g}^{2}$ ) where $G_{\mathrm{SNP}}$ was the SNP-derived GRM and $\sigma_{g}^{2}$ was the variance component of the random effect, the vector **b** was a random effect with distribution N(0, $G_{\mathrm{CNV}}{\text{σ}}_{c}^{2}$ ) where $G_{\mathrm{CNV}}$ was the CNV-derived GRM and ${\text{σ}}_{c}^{2}$ was the variance component of the random effect, and **e** was the random residual effect with distribution N(0, $D^{-1}{\text{σ}}_{e}^{2}$ ), where **D** was the diagonal matrix of weights on the deregressed EBVs for each animal and $\sigma_{e}^{2}$ was the residual variance component. The matrices **W** and **Z** were design matrices that related the random effects to each animal in the population.

Linear mixed models with a single random effect were also used to estimate the total additive genetic variance that can be explained by the SNP-derived GRM, the CNV-derived GRM, and the GRM derived from the combined SNP and CNV data. The fitted model was:

dEBV=μ+Za+e

where **dEBV** was a vector of deregressed EBVs, **μ** was the intercept term which was treated as a fixed effect, **a** was the polygenic random effect in the model which had the distribution N(0,**G** ${\text{σ}}_{a}^{2}$ ), where **G** represents either the SNP-derived GRM, the CNV-derived GRM, or the GRM derived from the combined SNP and CNV data, and the additive genetic variance component was ${\text{σ}}_{a}^{2}$ . The residual term **e** was treated as a random effect with distribution N(0, $D^{-1}{\text{σ}}_{e}^{2}$ ), the matrix **D** was a diagonal matrix with the weights of each deregressed EBV along the diagonal, and ${\text{σ}}_{e}^{2}$ was the residual variance component. The design matrix **Z** related the random effect to each animal in the population.

The equation used to calculate the weighting on each deregressed EBV is presented by Garrick et al. (2009):

$$w=\left(1-h^{2}²\right)/\left[c+\frac{1-r_{i}^{2}}{r_{i}^{2}}\right]h^{2}$$

The heritability of each trait was denoted by h^2^, the reliability of the EBV for the *i*th animal was given by r_i_^2^. The value of c, which was the proportion of genetic variation not explained by the model, was set to 0.9 for each trait as per Purfield et al. (2015).

A log-likelihood ratio test was used to compare the model with the two random genetic effects versus a model with only one random effect. The log-likelihood ratio test was done separately for each trait and breed to determine if the CNV-derived GRM can account for any variance in the random polygenic effect between animals. All mixed models were solved using the software suit ASREML 4.2 (Gilmour et al., 2015).

Separately, a two-step procedure was also undertaken to quantify the additive genetic variance of carcass traits attributable to the CNV-derived GRM after accounting for the effect of the SNP-derived GRM. The first step of this procedure was to model the deregressed EBVs using a linear mixed model in which the covariance structure of the random effect was reflected by the SNP-derived GRM. In the second step of this procedure, the residual term from this first step (after adding back the intercept values) was used as the dependent variable in a second linear mixed model. The fitted model was:

E=μ+Za+ε

where **E** is the sum of the residual and intercept term from the first model, **μ** was the intercept term which was treated as a fixed effect, **a** was the random effect vector which had the distribution N(0, $G_{\mathrm{CNV}}{\text{σ}}_{c}^{2}$ ) where $G_{\mathrm{CNV}}$ was the CNV-derived GRM and ${\text{σ}}_{c}^{2}$ was the variance component of the random effect, **Z** was a design matrix that related each animal in the dataset to the random effect, and **ε** was the random residual effect with distribution N(0, $D^{-1}{\text{σ}}_{\varepsilon }^{2}$ ), where **D** was a diagonal matrix in which the diagonal elements were the weights on the deregressed EBVs for each animal and ${\text{σ}}_{\varepsilon }^{2}$ was the residual variance component.

The variance component of the random effect of the second model, ${\text{σ}}_{c}^{2}$ , was the additive genetic variance attributable to CNV-derived GRM after accounting for the effect of the SNP-derived GRM**.**

### Association Analyses

A LASSO regression model was conducted to jointly evaluate the association between individual SNPs and CNVs and the deregressed carcass EBVs. The principal components of the SNP-derived GRM and the CNV-derived GRM were also included in the model as covariates to account for population stratification. The LASSO models were solved separately for each breed and trait using the R package “glmnet” (Friedman et al., 2010). Only the SNPs and CNVs with non-zero coefficients in the LASSO model were considered to be associated with the trait. The equation for the LASSO model was:

$$\beta^{lasso}=\text{minimize}\left\{\frac{1}{2N}\left\|{\left(y-\mathrm{X\beta}\right)}^{2}\right\|+\lambda \left\|\beta \right\|\right\}$$

The dependent variable, **y**, was the weighted deregressed EBVs for each animal. The matrix **X** consisted of the full set of SNPs and CNVs, the principal components the SNP and CNV GRMs, and an intercept term. The vector **β** was the effect of each independent variable in matrix **X**, λ was the penalty factor in the model, and the solutions, $\beta^{lasso},$ were obtained by minimising the prediction error of the general linear model with the penalty factor. The number of observations in the dataset was given by N and the notation ||.|| denotes the Euclidean norm.

Since none of the CNVs detected had at least 5 animals of a given breed carrying either a double-deletion or a single-deletion, the identified double-deletions were assumed as a single-deletion and were collapsed into a single deletion class. Similarly, all duplications were collapsed into a single duplication class and considered as single-duplications because there was no CNV with at least 5 animals within breed carrying either a double-duplication or a single-duplication. The number of animals and CNVs available for each breed and trait is in Table 1.

The LASSO model is a biased estimator of the regression coefficients for the associated genetic markers due to the inclusion of the penalty term in the LASSO model (Hastie et al., 2015). To obtain unbiased estimates of the regression coefficients, all of the genetic markers associated within breed for a particular trait were evaluated concurrently in a linear regression model without the penalty term used in the LASSO model; this process was carried out separately within breed for each trait analysed. This two-step procedure of identifying associated variants using the LASSO model and then obtaining unbiased estimates of the regression coefficients for the associated variants using simple linear regression is known as the relaxed-LASSO (Meinhausen, 2007; Hastie et al., 2015). The proportion of the genetic variance explained by associated CNVs was calculated separately for each CNV using the regression coefficients obtained from the unbiased model with the following equation:

$$2\left(p_{i}\right)\left(1-p_{i}\right)a_{i}^{2}/\sigma_{g}^{2}$$

where $p_{i}$ is the population frequency of the CNV as a proportion of the total population size, $a_{i}$ is the regression coefficient of the CNV (i.e., the allele substitution effect) taken from the unbiased linear model, and $\sigma_{g}^{2}$ is the within breed genetic variance for the carcass trait as used in the national genetic evaluation. The cumulative proportion of the genetic variance accounted for by the associated CNVs for each trait was calculated within breed by summing the proportion of the genetic variance attributable to each CNV associated with the carcass trait within breed.

To compare the results of the LASSO analysis with a more traditional genome-based association analysis, each of the associated variants identified by the LASSO analysis were individually re-analysed, within breed, using a linear mixed model approach. Along with an intercept term and the SNP genotype, the direct additive genetic effect of the animal was accounted for by fitting the SNP-derived GRM. The association of the genetic variant with the trait of interest was tested using a t-test under the null hypothesis that there was no association between the genetic variant and the trait of interest.

### Quantitative Trait Loci

For each associated SNP and CNV, a quantitative trait locus (QTL) region was designated as 50 kb upstream and 50 kb downstream of the SNP or CNV. When an associated SNP or CNV was located within 50 kb of another associated SNP or CNV, respectively, then they were merged into the same QTL; the boundary of these QTLs were 50 kb upstream and 50 kb downstream of the outermost variants in the QTL. The genomic position for each associated SNP and CNV were updated to the genomic positions given in the ARS-UCD1.2 assembly of the cattle genome (Rosen et al., 2020) in order to identify genes, as per the Ensembl bovine genome browser (http://ensembl.org), which overlapped in genomic position with each of the QTLs.

Gene set enrichment analysis was performed on the set of genes that overlapped with the QTL regions of each of the associated CNVs and SNPs identified by the LASSO regression analysis using the Database for Annotation, Visualisation, and Integrated Discovery (DAVID). This enrichment analysis was carried out separately per breed for the QTLs associated with each of the three traits. The DAVID algorithm identifies clusters of genes, assigns an enrichment score for the gene cluster, and gives a *p*-value for the observed enrichment score under the null hypothesis that there is no gene set enrichment.

## Results

The population structure of the entire population was examined using principal components analysis; the first principal component accounted for 6.2% of the total variance in the SNP-derived GRM and the second principal component accounted for 3.3% of the total variance in the SNP-derived GRM (Supplementary Figure S1). The top principal component of SNP-derived GRM for the Holstein-Friesians accounted for approximately 4.1% of the total variance. This was more than three times greater than the percentage variance explained by the top principal component of the SNP-derived GRMs for either the Charolais or the Limousin populations.

### Variance Components Analysis

After edits 13,969, 3,954, and 2,805 CNVs were available to calculate the CNV-derived GRMs for the Holstein-Friesian, Charolais, and Limousin populations, respectively, whereas for each breed, 712,555 SNPs were used to calculate the SNP-derived GRMs. For each breed, the CNV-derived GRM was calculated twice using the two separate procedure described in the methods. The variance components analysis was conducted separately for these two sets of CNV-derived GRMs; there was no difference in the results of the variance components analysis using either of these two sets of CNV-derived GRMs. The proportion of the variance accounted for by the SNP-derived GRM when considered in a linear mixed model was between 0.032 and 0.291 for the three carcass traits analysed in the three breeds (Supplementary Table S1). In comparison, when considered singly, the CNV-derived GRM explained almost none of the variance in the deregressed EBVs; this was the case for all traits in all breeds (Supplementary Table S1). When the CNV-derived GRM and SNP-derived GRM were considered jointly in the same model, the CNV-derived GRM did not account for any of the variance in the deregressed EBVs for any trait in all three breeds. The marginal contribution of the CNV-derived GRM to the additive genetic variance of the carcass traits was also calculated using a stepwise approach in which the CNV-derived GRM was used to model the random polygenic effect of each animal after accounting for the effect of the SNP-derived GRM. The conclusion from this approach was the same as that from the joint evaluation of the CNV-derived GRM and SNP-derived GRM. Similarly, for all traits and all breeds, no difference in fit existed between a model that accounted for the polygenic random effect using only the SNP-derived GRM, or a model which accounted for the polygenic random effect using a single GRM derived from the combined SNP and CNV datasets.

### Association Analyses

A total of 987 SNPs and 18 CNVs were associated with at least one of the three carcass traits in at least one of the three breeds analysed. The number of CNVs and SNPs associated with each of the three carcass traits per breed is given Table 1. None of the 18 associated CNVs were located within 50 kb of another associated CNV; hence there were 18 distinct CNV QTLs. The 987 SNPs formed 699 distinct SNP QTLs. One QTL harboured both an associated SNP and an associated CNV; therefore, overall 717 QTLs were identified in the present study. There was no gene set enrichment for any biological functions among the sets of genes which overlapped with the QTLs associated with each of the carcass traits within breed. A list of all 18 CNV QTLs is given in Table 2; 15 of the 18 CNV QTLs overlapped with a previously reported CNV region as per the Ensembl bovine genome browser. For each of the three carcass traits, the associated CNVs identified by the LASSO regression analysis cumulatively accounted for between 0.000% (carcass weight in the Holstein-Friesians) and 1.236% (carcass weight in the Charolais) of the total genetic variance. The genetic variance explained by the associated CNVs for each of the three carcass traits within breed is in Table 1. None of the CNVs were associated with more than one trait within breed or across breeds. Furthermore, none of the CNVs were associated with the same trait in more than one breed even though 10 of the 18 associated CNVs were present in more than one breed.

**TABLE 2:** Chromosome, position, and candidate gene(s) for each copy number variant (CNV) quantitative trait locus (QTL) associated with each of the carcass traits in Charolais (CH), Holstein-Friesian (HF), and Limousin (LM). When no genes overlapped the QTL region none is reported in the candidate gene column.

Breed	Trait	Chromosome	QTL start, mb	QTL end, mb	Candidate Genes
CH	Conformation	1	102.44	102.46	*U6*
CH	Conformation	12	37.44	37.44	None
CH	Conformation	15	83.77	83.80	None
CH	Fat	2	123.74	123.79	None
CH	Fat	4	54.37	54.40	None
CH	Fat	5	80.51	80.53	None
CH	Fat	9	2.55	2.57	None
CH	Fat	12	57.90	57.91	None
CH	Fat	17	71.37	71.39	*CABIN1*
CH	Fat	17	71.70	71.74	*BCR, SPECC1L*
CH	Fat	25	15.49	15.50	*XYLT1*
CH	Fat	29	36.70	36.77	*ADAMTS8, ADAMTS15,*
CH	Fat	12	60.28	60.28	None
CH	Weight	4	83.86	83.87	*5s_rRNA*
CH	Weight	9	7.13	7.14	None
HF	Conformation	20	42.99	43.02	None
LM	Conformation	7	43.87	43.90	*REEP6, PCSK4, APC2, ADAMTSL5*
LM	Fat	10	39.64	39.65	*RPL10L*

The QTL with an associated CNV and an associated SNP was associated with carcass fat in the Charolais and was located on chromosome 29 between 36.70 and 36.77 Mb. The CNV in this QTL was a duplication present in 8 animals and the associated SNP in this QTL, rs109364153, was located within the genomic position of the associated CNV; this QTL overlapped in genomic position with a single gene which was the RNA gene *bta-mir-584-7*. Single nucleotide polymorphisms close to this QTL region were also associated with carcass conformation and weight in the Limousins.

**FIGURE 1:**
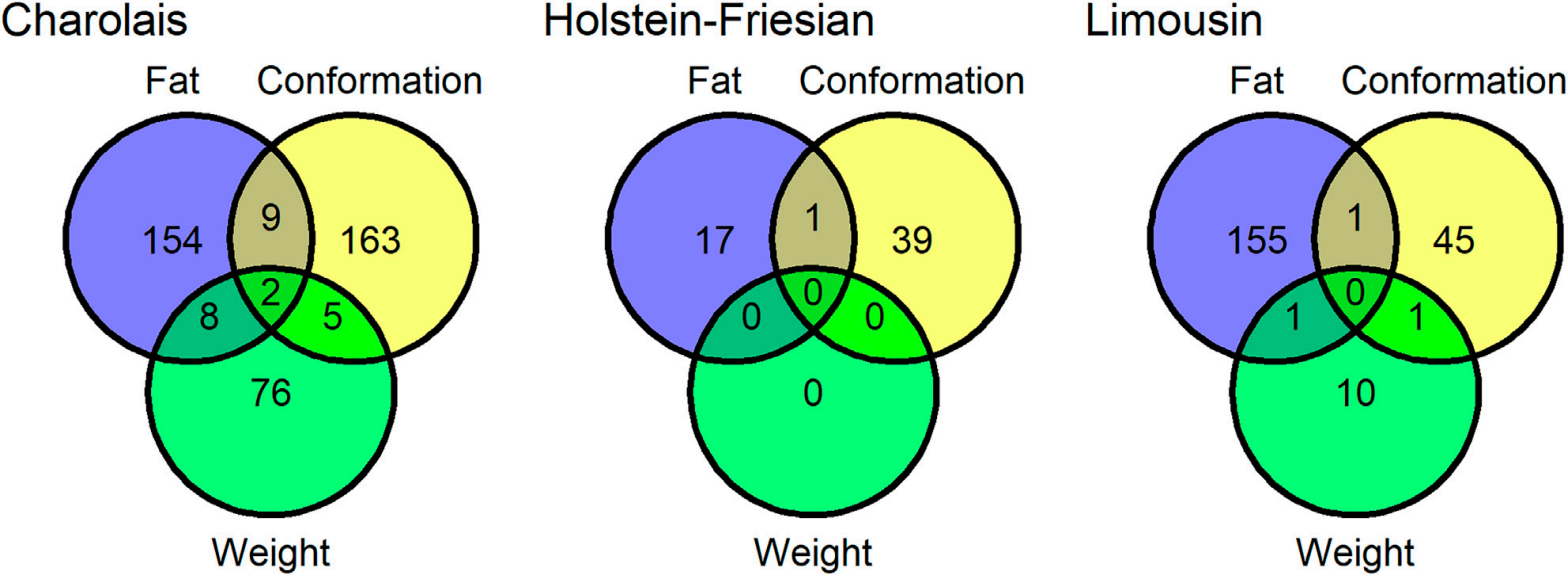
The number quantitative trait loci (QTLs) associated with carcass conformation, carcass fat and carcass weight in the Charolais, Holstein-Friesians, and Limousins. Quantitative trait loci that overlapped in genomic position with another QTL associated with a different trait in the same breed were considered a single QTL shared between the two traits.

**FIGURE 2:**
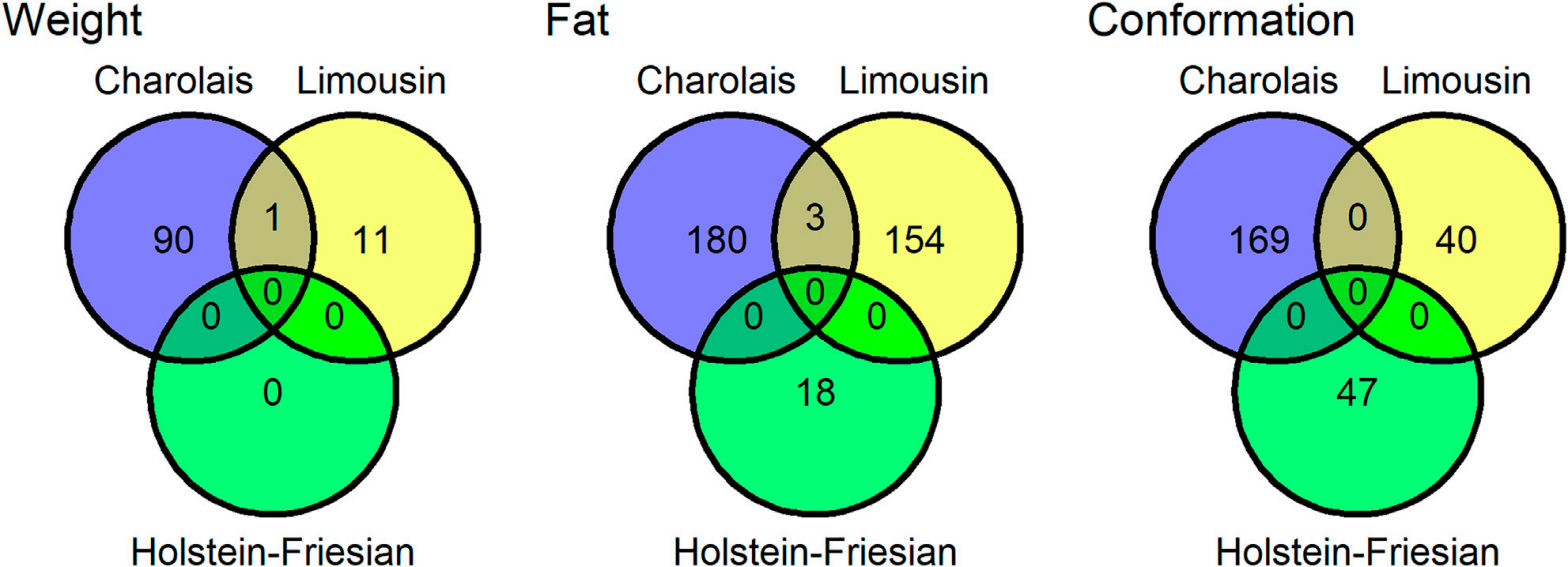
The number quantitative trait loci (QTLs) associated with carcass weight, carcass fat, and carcass conformation for the Charolais, Holstein-Friesian, and Limousin populations. Quantitative trait loci that overlapped in genomic position with another QTL associated with the same trait in another breed were considered a single QTL shared between the two breeds.

Twenty-eight SNP QTLs were associated with more than one trait within breed (Figure 1) and another 4 SNP QTLs were associated with the same trait in more than one breed (Figure 2). In the Charolais, the QTL with largest number of associated SNPs contained 8 SNPs associated with carcass fat. This QTL was located on chromosome 2 between 6.25 and 6.29 Mb and SNPs within this genomic region were also associated with carcass conformation and carcass weight in the Charolais. Similarly in the Limousins, a SNP QTL also associated with carcass fat was located on chromosome 2 between 6.14 and 6.25 Mb and contained 18 associated SNPs. In the Limousins, this QTL had the greatest number of SNPs associated with any trait; SNPs in the genomic region of this QTL were also associated with carcass conformation in the Limousins. The protein coding gene *MSTN* is the most likely candidate gene for these two QTLs identified in the Limousins and the Charolais*.* None of the 26 SNPs in the genomic region of these two QTLs were associated with any of the three carcass traits in the Holstein-Friesians, even though each of the 26 SNPs were segregating in the Holstein-Friesians. A list of the top five SNP QTLs associated with each of the three carcass traits within breed is given in Supplementary Tables S2–S4.

All of the associated SNPs and CNVs were re-analysed singly using a linear mixed model approach; the nominal *p*-values for each of the tested variants was between 0.027 and 1.4 × 10^−15^ (Supplementary Figure S2).

## Discussion

Copy number variants have been documented to be associated with several performance traits in cattle such as meat quality (da Silva et al., 2016), carcass merit (Zhou et al., 2016), and milk yield (Xu et al., 2014; Prinsen et al., 2017). While these studies demonstrate that CNVs can be used to identify genomic regions associated with important traits in cattle, it is often uncertain whether CNVs uniquely identify these genomic regions or if SNP genotype data could have been used to detect the same genomic regions. Based on a population of 2,230 Nellore cattle, Hay et al. (2018) performed the first genomic predictions of carcass traits in cattle that used both SNP and CNV data; they found that, for some traits, the CNV data improved the accuracy of genomic predictions. However, it is still unclear how additive genetic variance is partitioned between CNVs and SNPs, or if CNV data can identify genomic regions in a genome-wide association analysis that would otherwise be undetected if SNP genotypes were the only genetic markers used in the analysis.

Based on the top two principal components of the SNP-derived GRM for the entire population (Supplementary Figure S1), two closely related clusters of animals existed in the Holstein-Friesian population, whereas for the Charolais and Limousin populations each breed consisted of only a single cluster of animals. This indicated that the Holstein-Friesian population in the present study was less homogeneous than either the Charolais or the Limousin populations which is not unexpected given that both Holstein and Friesian founder animals will have contributed to the Holstein-Friesian population in the present study. This may account for the greater number of observed CNVs in the Holstein-Friesian population when compared to the Charolais or the Limousin populations.

### Copy Number Variant Association Analysis

Although the CNV-derived GRM could not account for any additive genetic variation in the three carcass traits, the LASSO regression model identified 18 CNVs associated with at least one of the three carcass traits across the three breeds analysed. While the results of LASSO analysis and the CNV-derived GRM analysis appear to be in opposition, in reality the associated CNVs identified by the LASSO regression analysis only explained between 0.000 and 1.236% of the total genetic variance for each of the three carcass traits analysed (Table 1). So the actual difference in the genetic variance explained by the variance components analysis and the LASSO regression analysis was small. Considering that the proportion of the genetic variance explained by the CNVs was small, it may be the case that a CNV-derived GRM is unable to detect this genetic variance when jointly evaluated in a model with a SNP-derived GRM. This may also explain why Hay et al. (2018) found that exploiting CNV information improved the accuracy of genomic predictions for some carcass traits because, in that study, the CNVs were modelled as fixed effects in the genomic prediction models. The LASSO model might be better suited at detecting the genetic variance explained by CNVs because CNVs which tend to have low population frequency (Supplementary Figure S3) and low reproducibility may be preferentially removed from the LASSO model. Genetic markers with low population frequencies have less power to detect associations in an association analysis (Bush and Moore, 2012) and errors in genotype assignment or CNV copy number assignment also reduce the power to detect associations when the error is independent of the phenotypic value (Pompanon et al., 2005; Cook et al., 2014; Hou et al., 2017). In a LASSO model, genetic markers with no detectable effect tend to be eliminated from the model (Hastie et al., 2015); therefore those CNVs which have no detectable effect should be preferentially removed from the LASSO model. Hence the LASSO model may be better suited to accounting for the genetic variance explained by CNVs rather than the variance components analysis.

Several of the associated CNVs overlapped in genomic position with genes and CNVs that have previously been associated with carcass traits in cattle. For instance, a deletion present in 3 animals in the Charolais, located on chromosome 5 between 80.51 and 80.76 Mb, was approximately 170 kb from *FAR2. FAR2* is a gene related to fatty acid synthesis in mammals (Cheng et al., 2004) and has been associated with meat quality in Hanwoo cattle (Lim et al., 2016). A duplication CNV present in 4 animals in the Charolais population of the present study was associated with carcass fat and was located approximately 390 kb from the RNA gene *Metazoa_SRP.* Differential expression of this gene in Qinchuan cattle is related to the development of skeletal muscle (Li et al., 2016). A CNV located on chromosome 17 between 71.37 Mb and 71.39 Mb was associated with carcass fat in the Charolais and overlapped with CNVs identified in another study that were associated with stature in Holstein cattle (Sassi et al., 2016). The QTL on chromosome 17 between 71.37 Mb and 71.39 Mb was located approximately 310 kb from the protein coding gene *PRAME* which has previously been associated with carcass yield in a CNV association study (Zhou et al., 2016). The CNV on chromosome 17 between 71.37 Mb and 71.39 Mb was also located 340 kb from a second CNV between 71.70 Mb and 71.74 Mb which was also associated with carcass fat in the Charolais in the present study; both CNVs were called in different cohorts of animals. Furthermore, no other CNVs existed in the Charolais, Holstein-Friesian, or Limousin populations which overlapped the genomic position of both CNVs. This suggests that these two CNVs, despite being in close genomic proximity, were two distinct CNV regions. Given that some of the associated CNVs detected in the present study overlapped with (or were flanked by) genes and CNVs associated with carcass and related traits in cattle, it suggests that at least some of the 18 associated CNVs were true associations.

In addition to these genes which have previously been associated with carcass traits in cattle, several novel candidate genes also overlapped with CNV QTLs. The protein coding gene *PTPRU* was located approximately 450 kb from a CNV QTL on chromosome 2 between 123.74 and 123.79 Mb; this CNV was associated with carcass fat in the Charolais and none of the associated SNPs identified in the present study overlapped in genomic position with this QTL. This gene has not previously been associated with carcass traits in cattle although it has been associated with feed conversion rate in pigs (Horodyska et al., 2017) and wither height in Yaks (Jia et al., 2019).

### Single Nucleotide Polymorphism Association Analysis

The fact that the Charolais and the Limousins shared more QTLs than either breed shared with the Holstein-Friesians substantiates the known shared genetic ancestry of these breeds (Bouquet et al., 2011; Kelleher et al., 2017). Several of the QTLs that were shared between breeds also overlapped in genomic position with genes which have previously been associated with carcass traits in cattle. The protein coding gene *RSAD2* overlapped in genomic position with a SNP QTL located on chromosome 11 at 90.11 Mb which was associated with carcass fat in the Charolais and the Limousins. This gene has previously been associated with carcass traits in Nellore cattle (Silva-Vignato et al., 2017) and width of withers in Hereford cattle (Doyle et al., 2020).

In addition to the candidate genes which have previously been identified in genome-wide association studies of carcass traits in cattle, several novel candidate genes were also identified in the present study. The protein coding gene *USP24* was located in close genomic proximity (<370 kb) to two SNP QTLs. The first SNP QTL was located on chromosome 3 between 90.89 and 90.90 Mb and was associated with carcass conformation in the Charolais; the second SNP QTL was located on chromosome 3 at 90.76 Mb and was associated carcass conformation in the Limousins. This gene has previously been associated with milk yield and mastitis resistance in cattle (Cai et al., 2020) and growth traits in pigs (Xiong et al., 2015), but has not previously been associated with carcass traits in cattle. The protein coding gene *ACTN2* was located 230 kb from a SNP positioned on chromosome 28 at 9.09 Mb which was associated with carcass fat in the Charolais. *ACTN2* also overlapped with a SNP on chromosome 28 at 9.35 Mb which was associated with carcass fat in the Limousins. In mammals, *ACTN2* is highly expressed in skeletal and cardiac muscle (Lornage et al., 2019) and mutations in *ACTN2* cause cardiac myopathy (Lornage et al., 2019) and muscular dystrophy (Lornage et al., 2019; Savarese et al., 2019) in humans. Another novel candidate gene identified in the present study was *THOC1*; this gene overlapped with a SNP QTL on chromosome 24 between 35.22 and 35.25 Mb which was associated with carcass fat in the Charolais and Limousins. *THOC1* may have a role in muscle cell proliferation in pigs (Zappaterra et al., 2020).

The 28 SNP QTLs which were associated with more than one trait within breed may be contributing to the genetic correlation between carcass traits in cattle (Hickey et al., 2007; Kause et al., 2015). Two SNP QTLs were associated with all three carcass traits within a single breed, possibly contributing to the genetic correlation between all three traits. *MSTN* overlapped with a SNP QTL that was associated with all three traits in the Charolais as well as carcass fat and conformation in the Limousins. *MSTN* is known to have a large effect on carcass traits in cattle (Grobet et al., 1997; Kambadur et al., 1997; McPherron et al., 1997).

The dataset used in the present study had previously been used as part of a larger dataset which consisted of 28,470 sires across six different breeds with each of the sires imputed to 41,389,526 SNPs. This dataset was used by Purfield et al. (2019) to perform a traditional genome-based association analysis of carcass traits in cattle. A total of 55 SNP QTLs (7.7% of all QTLs) identified in the present study overlapped in genomic position with the QTLs identified by Purfield et al. (2019). In Purfield et al. (2019), as in the present study, the associated SNPs located near *MSTN*, *NCAPG,* and *LCORL* were associated with at least one of the carcass traits in the Charolais and the Limousin populations. Purfield et al. (2019) identified only one SNP QTL which was associated with carcass weight in the Holstein-Friesians; this SNP QTL was located on chromosome 14 between 24.49 and 25.33 Mb. This result was similar to the present study in which no SNPs or CNVs were associated with carcass weight in the Holstein-Friesians.

## Conclusion

The CNV-derived GRM did not account for any additive genetic variance in the three carcass traits when jointly evaluated with a SNP-derived GRM in a linear mixed model for each of the three breeds. A LASSO regression analysis which jointly evaluated SNPs and CNVs did, however, identify 18 CNVs associated with at least one of the three carcass traits in the three breeds analysed although the proportion of genetic variance explained was small. Some of these associated CNVs overlapped with genes that have previously been associated with carcass traits, which suggests that these CNVs represent true associations. In addition to reaffirming candidate genes that have previously been associated with carcass traits in cattle, several novel candidate genes where also identified. Novel candidate genes which overlapped with CNV QTLs included *PTPRU* and novel candidate genes which overlapped with SNP QTLs included *ACTN2, THOC1,* and *USP24.*

## Data Availability

The data analyzed in this study is subject to the following licenses/restrictions: The phenotype and genotype data analyzed in the present study are the property of the Irish Cattle Breeding Federation (ICBF). Requests to access these datasets should be directed to DB, donagh.berry@teagasc.ie.
