# Supplementary material for: The Contribution of Copy Number Variants and Single Nucleotide Polymorphisms to the Additive Genetic Variance of Carcass Traits in Cattle
Source: Front Genet. 2021 Nov 2;12:761503. doi: 10.3389/fgene.2021.761503 (PMC8593468; doi:10.3389/fgene.2021.761503)
Supplement: Supplementary file 1 [file Presentation1.zip › Suppl. Figure 1.docx]

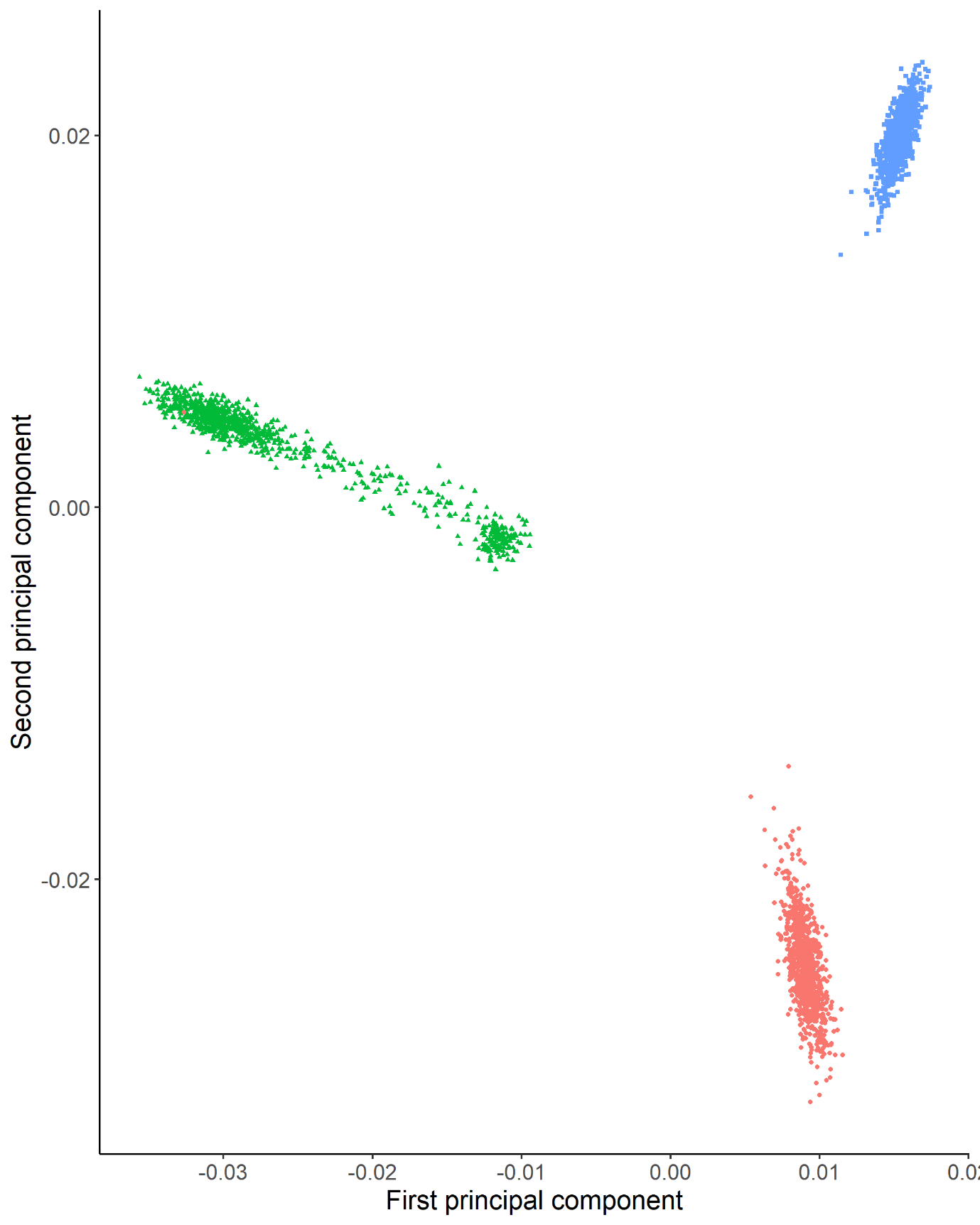


Figure S1. Principal components analysis of all available animals in the population (n=2,856). The Charolais animals (n=945) were represented by red circles, the Holstein-Friesian animals (n=923) were represented by green triangles, and the Limousin animals (n=974) were represented by blue squares.
