# Supplementary material for: The Contribution of Copy Number Variants and Single Nucleotide Polymorphisms to the Additive Genetic Variance of Carcass Traits in Cattle
Source: Front Genet. 2021 Nov 2;12:761503. doi: 10.3389/fgene.2021.761503 (PMC8593468; doi:10.3389/fgene.2021.761503)
Supplement: Supplementary file 1 [file Presentation1.zip › Suppl. Figure 2.docx]

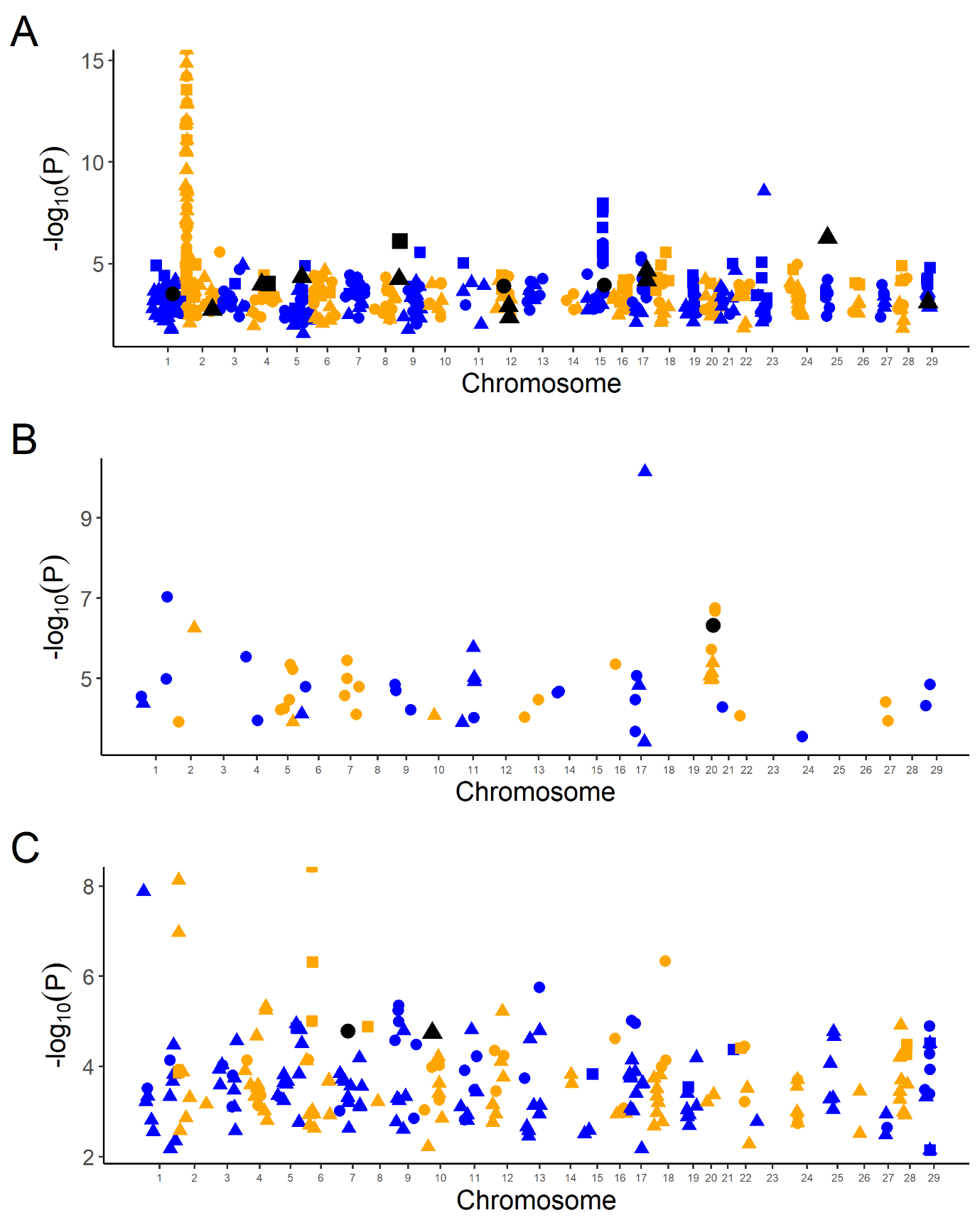


Figure S2. Manhattan plot of the associated single nucleotide polymorphisms (SNPs) and copy number variants (CNVs) for the A) Charolais, B) Holstein-Friesians, and C) Limousins. Genetic variants associated with carcass weight are represented by squares, genetic variants associated with carcass fat are represented by circles, and genetic variants associated with carcass conformation are represented by triangles. Copy number variants were coloured black.
