# Supplementary material for: The Contribution of Copy Number Variants and Single Nucleotide Polymorphisms to the Additive Genetic Variance of Carcass Traits in Cattle
Source: Front Genet. 2021 Nov 2;12:761503. doi: 10.3389/fgene.2021.761503 (PMC8593468; doi:10.3389/fgene.2021.761503)
Supplement: Supplementary file 1 [file Presentation1.zip › Suppl. Figure 3.docx]

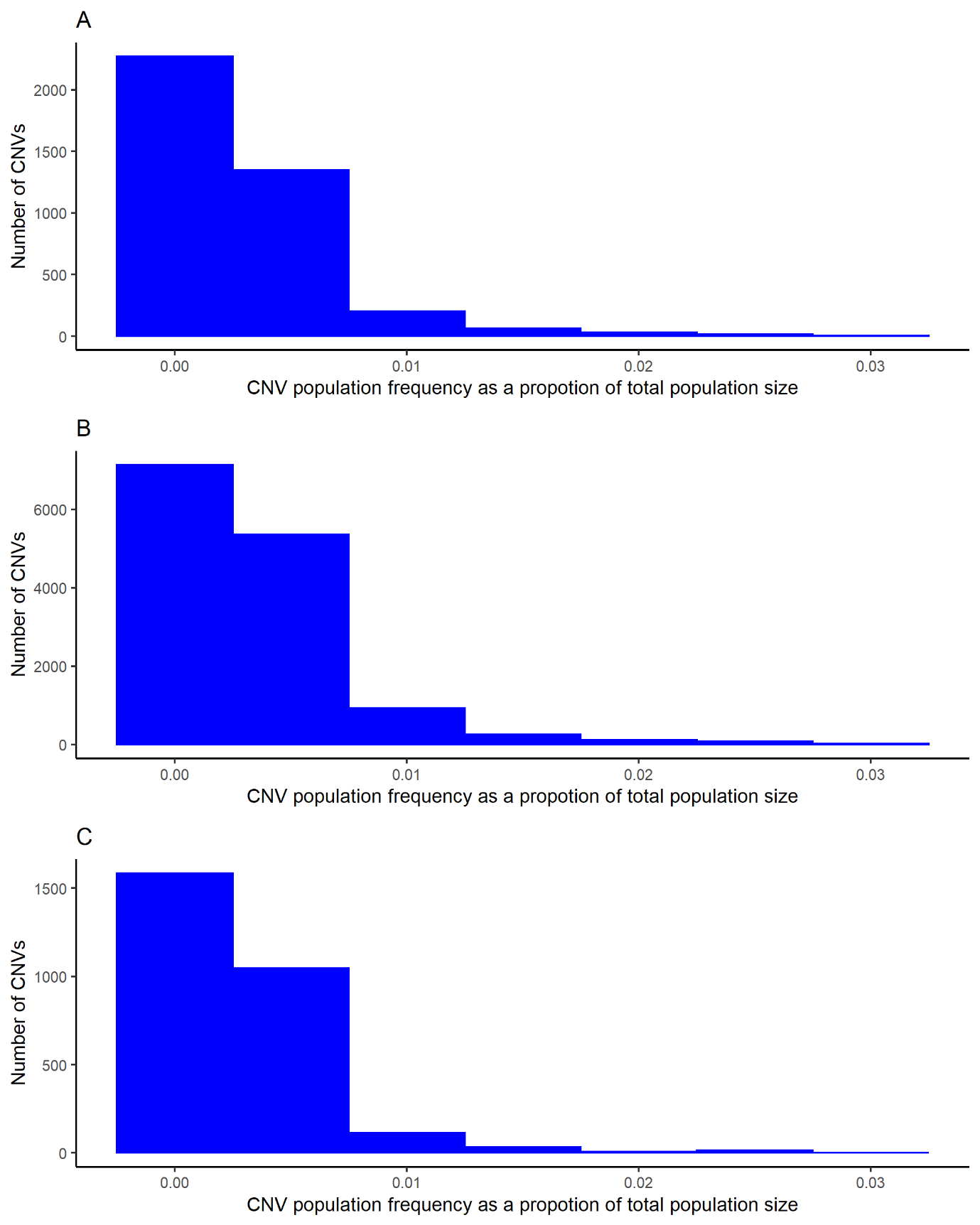


Figure S3: Histogram of the population frequency of the available copy number variants (CNVs) as a proportion of the total population size in the A) Charolais, B) Holstein-Friesian, C) Limousin. Copy number variants which were present in more than 3% of animals within breed were excluded. In total 18 CNVs were excluded from the Charolais, 82 CNVs were excluded from the Holstein-Friesians, and 6 CNVs were excluded from the Limousins.
