# Supplementary material for: The Contribution of Copy Number Variants and Single Nucleotide Polymorphisms to the Additive Genetic Variance of Carcass Traits in Cattle
Source: Front Genet. 2021 Nov 2;12:761503. doi: 10.3389/fgene.2021.761503 (PMC8593468; doi:10.3389/fgene.2021.761503)
Supplement: Supplementary file 2 [file Table1.docx]

Table S1: The proportion of the variance accounted for the SNP-derived genetic relationship matrix (GRM) and the proportion of the variance accounted for by the CNV-derived GRM when separately and singly considered in a linear mixed model for all traits in all breeds analysed.

| Breed | Trait | SNP GRM variance proportion | CNV GRM variance proportion |
| --- | --- | --- | --- |
| Charolais | Weight | 0.196 | 2.7x10^-7^ |
| Charolais | Fat | 0.288 | 1.6x10^-7^ |
| Charolais | Conformation | 0.291 | 2.0x10^-7^ |
| Holstein-Friesian | Weight | 0.032 | 7.7x10^-7^ |
| Holstein-Friesian | Fat | 0.097 | 7.7x10^-7^ |
| Holstein-Friesian | Conformation | 0.065 | 7.7x10^-7^ |
| Limousin | Weight | 0.145 | 1.1x10^-7^ |
| Limousin | Fat | 0.244 | 8.1x10^-7^ |
| Limousin | Conformation | 0.286 | 7.1x10^-3^ |
