# Supplementary material for: The Contribution of Copy Number Variants and Single Nucleotide Polymorphisms to the Additive Genetic Variance of Carcass Traits in Cattle
Source: Front Genet. 2021 Nov 2;12:761503. doi: 10.3389/fgene.2021.761503 (PMC8593468; doi:10.3389/fgene.2021.761503)
Supplement: Supplementary file 5 [file Table4.docx]

Table S4. The chromosome, position, number of associated single nucleotide polymorphism (SNPs), and candidate gene for of the top five SNP quantitative trait loci (QTL) for each trait in the Limousin.

| Trait | Chromosome | QTL Start, Mb | QTL End, Mb | Candidate  genes | Number of SNPs |
| --- | --- | --- | --- | --- | --- |
| Conformation | 3 | 47.19 | 47.22 | *-* | 4 |
| Conformation | 5 | 4.84 | 4.89 | *GLIPR1L1* | 3 |
| Conformation | 9 | 21.56 | 21.57 | *TENT5A* | 3 |
| Conformation | 22 | 35.05 | 35.06 | *LRIG1* | 3 |
| Conformation | 29 | 36.15 | 36.16 | *PRDM10* | 2 |
| Fat | 2 | 6.14 | 6.25 | *MSTN* | 18 |
| Fat | 2 | 5.96 | 6.05 | *MSTN* | 8 |
| Fat | 4 | 68.43 | 68.47 | *HIBADH* | 6 |
| Fat | 19 | 57.48 | 57.53 | *-* | 6 |
| Fat | 5 | 98.47 | 98.54 | *TAS2R42* | 5 |
| Weight | 28 | 37.92 | 37.94 | *NRG3* | 2 |
| Weight | 6 | 37.14 | 37.14 | *NCAPG, LCORL* | 1 |
| Weight | 29 | 38.09 | 38.09 | *PAG15, PAG7* | 1 |
| Weight | 2 | 7.48 | 7.48 | *MSTN* | 1 |
| Weight | 5 | 86.58 | 86.58 | *SOX5* | 1 |
